# Determinants of maternal low mid‐upper arm circumference and its association with child nutritional status among poor and very poor households in rural Bangladesh

**DOI:** 10.1111/mcn.13217

**Published:** 2021-05-20

**Authors:** Md Ahshanul Haque, Nuzhat Choudhury, Fahmida Dil Farzana, Mohammad Ali, Mohammad Jyoti Raihan, S. M. Tanvir Ahmed, Sheikh Shahed Rahman, Towfida Jahan Siddiqua, Abu Syed Golam Faruque, Tahmeed Ahmed

**Affiliations:** ^1^ Nutrition and Clinical Services Division icddr,b Dhaka Bangladesh; ^2^ Child Poverty Sector Save the Children Bangladesh Dhaka Bangladesh

**Keywords:** anthropometry, Bangladesh, baseline survey, child nutrition, food insecurity, maternal nutrition, mid‐upper arm circumference, public health, Suchana, underweight

## Abstract

Malnutrition among women is a long‐standing public health concern that has significant adverse consequences on the survival and healthy development of children. Maternal mid‐upper arm circumference (MUAC) could potentially represent a simpler alternative to traditional nutritional indicators. This study aimed to investigate the factors associated with low maternal MUAC (as an indicator of being underweight) and address the research question of whether maternal MUAC is significantly associated with children's nutritional status among poor and very poor households in rural Bangladesh. Data on 5,069 households were extracted from the Suchana programme baseline survey, which was carried out in 80 randomly selected unions (the lowest administrative unit of Bangladesh) in Sylhet and Moulvibazar districts between November 2016 and February 2017. The outcome variables were three child nutritional status indicators: wasting, stunting and underweight. Mothers were classified as underweight if their MUAC was less than 23 cm. Separate multiple logistic regression analyses were used to determine the factors potentially associated with maternal underweight status and explore whether maternal underweight status is significantly associated with children's nutritional status. The prevalence of maternal underweight status was 46.7%, and the prevalence of wasting, stunting and underweight among children under two were 10.5%, 44.4% and 31.9%, respectively. After controlling for various socio‐economic and demographic characteristics, maternal MUAC was significantly associated with children's nutritional status in rural Bangladesh.

**Key messages**

- Low maternal MUAC may indicate worsening nutritional status among children.
- To reduce maternal underweight status, public health strategies should focus on water, sanitation and hygiene (WASH), household food insecurity and optimal maternal healthcare practices.
- MUAC measuring tapes are cheap and widely available and enable underweight women to be easily identified in low‐resource settings.

## 1 INTRODUCTION

Women of child‐bearing age are often nutritionally susceptible (Lartey, 2008). Although maternal and child nutrition have improved much globally, the prevalence of maternal and child malnutrition still remains very high in low‐resource settings. Despite the successful implementation of many nutritional policies at the national and international level, maternal malnutrition still represents a major public health concern that has adverse consequences on the survival and healthy advancement of women, especially in low‐ and middle‐income countries (Black et al., 2013; National Institute of Population Research and Training ‐ NIPORT/Bangladesh, Mitra and Associates, & ICF International, 2016). Previous studies have shown maternal malnutrition is commonly associated with childhood malnutrition and contributes to low birth weight, suggesting new avenues of research and novel interventions are required to reduce maternal and child malnutrition (Ajieroh, 2009; Bhutta et al., 2008; Choudhury et al., 2017; Islam et al., 1994; Lartey, 2008).

The situation of maternal undernutrition in Bangladesh is no better than in other low‐ and middle‐income countries. The Food Security Nutritional Surveillance Project reported that 18% of women are undernourished nationally based on mid‐upper arm circumference (MUAC) measurements, with an alarmingly high prevalence of 40% in Sylhet region (HKI and JPGSPH, 2014). Sylhet is one of the most vulnerable regions in northeast Bangladesh and includes diverse terrain such as plains, hills, *Haor* (wetlands) and areas prone to flash flooding (HKI and JPGSPH, 2014). Several important indicators of healthcare, maternal nutrition and socio‐economic status are low in this region of the country (HKI and JPGSPH, 2014; NIPORT/Bangladesh et al., 2016). In contrast to the national decrease observed in Bangladesh overall, child malnutrition has increased in Sylhet in recent years (NIPORT/Bangladesh et al., 2016).

Numerous factors are associated with women's undernutrition, including socio‐economic and demographic characteristics, smoking habits, household food insecurity status, decision‐making autonomy and the area of residence (Balarajan & Villamor, 2009; Corsi et al., 2011; Hasan et al., 2017; Kamal et al., 2015; Khan & Kraemer, 2009). Nationally representative studies in Bangladesh have reported associations between child or maternal malnutrition and the wealth index, which is a composite indicator of several household socio‐economic characteristics (NIPORT/Bangladesh et al., 2016). However, it remains unclear whether specific socio‐economic indicators—such as the type of latrine, source of drinking water, household construction materials or the number of children—influence the nutritional status of women, especially those from poor and very poor households.

Household food security status is a critical factor associated with childhood malnutrition that contributes to maternal malnutrition (S. L. Young et al., 2014). However, only a small number of studies have tested this association nationally or among the general Bangladeshi population, and no studies have been conducted among vulnerable populations living in specific regions of Bangladesh. In addition to the household food security status, biological factors also affect child malnutrition, and the mother plays a significant role in the interaction between children and the environment (Victora et al., 2008). Intrauterine nutritional deficiency and a lack of handwashing reduce the energy balance of the child (Sawaya & Roberts, 2003), which may have harmful effects during the late prenatal stages and lead to short height due to long‐term nutritional deficits (Victora et al., 2008). However, many other factors have also been associated with maternal and child nutrition, including poor maternal reproductive healthcare‐related indicators, experience of domestic violence and a lack of handwashing with soap (Adhikari et al., 2020; Chattopadhyay et al., 2019; Nguyen et al., 2017; Nkoka et al., 2019).

The nutritional indicator body mass index (BMI) is calculated using weight (kg)/height (m)^2^ (Ajieroh, 2009; Choudhury et al., 2017; Islam et al., 1994; Lartey, 2008; NIPORT/Bangladesh et al., 2016). However, there are some practical limitations to the use of BMI as a rapid assessment tool, as it can be difficult to measure the height and weight of debilitated, disabled or acutely ill individuals. Moreover, the instruments used to measure weight and height/length are not always precise. High‐precision instruments are very costly in resource‐poor settings or population‐based surveys and require trained personnel, which is not always possible. MUAC represents a sensible alternative in such situations and is the most common anthropometric measure used to assess the nutritional status of children (Briend et al., 1987; Sultana et al., 2015; WHO Working Group, 1986), particularly in emergency and crisis settings. Therefore, MUAC is considered as a reliable method for determining the nutritional status of women (Sultana et al., 2015). Several studies have been conducted to assess the association between maternal MUAC and children's nutritional indicators in various settings and populations (Assefa et al., 2012; Gewa et al., 2012; Kpewou et al., 2020; Vasundhara et al., 2020). These studies measured the nutritional status of either lactating or pregnant women (representing the general population) in facilities or households but did not specifically focus on women from poor or very poor households. Thus, there is a lack of research on the relationships between maternal underweight status determined using MUAC and the multiple factors mentioned above in rural Bangladesh, particularly among women belonging to vulnerable households.

To address this knowledge gap, this paper aimed to identify the factors associated with maternal MUAC and investigate whether these indicators are significantly associated with children's nutritional status among a socio‐economically deprived population in rural Bangladesh. To our knowledge, this is the first study of maternal MUAC among poor and very poor communities. The results of this study are expected to inform the formulation of appropriate strategies to reduce the worsening nutritional status of babies yet to be born in the Sylhet region of Bangladesh based on the MUAC of pregnant women.

## 2 METHODS

### 2.1 Source of data

Data for this study were extracted from the baseline cross‐sectional survey of the Suchana programme, a large‐scale development programme that is currently ongoing in Sylhet division (Choudhury et al., 2020; Haque et al., 2020). The baseline survey was conducted between November 2016 and February 2017 and covered 640 villages from 80 unions (the smallest administrative unit) in the northeast of Bangladesh (HKI and JPGSPH, 2014). The 80 unions were randomly selected from a list of the 157 unions in Moulvibazar and Sylhet districts of Sylhet division. For implementation purposes, vulnerable villages in each union were selected by the programme staff based on vulnerability (e.g., frequency of flooding/submerging, low or no intervention by other development programmes, poverty/household living conditions, remoteness/difficulty to reach and superstitions/high prevalence of social taboos). This selection process was finalized after discussion with local government officials, elected representatives, local elites and field visits. Eight vulnerable villages were randomly selected from each union for the baseline survey.

A household wealth ranking was completed; all listed households were verified as per the Suchana programme inclusion criteria and, if selected, given an identification number. Finally, households with a mother–child pair were selected for the baseline survey using a systematic sampling method (Choudhury et al., 2020); the age range for mothers was 15–49 years and children 0–23 months. If any selected households were unavailable due to non‐response or other reasons (Figure S1), we replaced the household with the next immediate household from the sampling frame in an anti‐clockwise direction (Haque et al., 2020) to survey the required number of households by phase and age group, according to our randomly generated listing. In this analysis, cases were excluded when the maternal age was <20 years.

Survey data collection was conducted using Android tablets complemented with custom‐developed Java software. Separate standard operating procedures were followed for interviewing and anthropometry. The survey team underwent 40 days of training, including field‐testing and adjustment of the data collection tools. The training included a brief programme overview; general rules, norms and guidance on survey implementation; the survey methodology, such as team composition, sampling and household selection process; a detailed discussion of the questionnaire form, interview techniques, administration of questionnaires and handling the Android PDAs; role play exercises aided by demonstrations to better understand techniques for asking sensitive questions and methods of ensuring the quality of the data. The dedicated anthropometry team was trained on anthropometric measurements and how to assemble, calibrate and maintain their tools, as well as the key points to check while taking measurements. A master trainer was involved in training the anthropometric team.

### 2.2 Variables of interest

**FIGURE 1:**
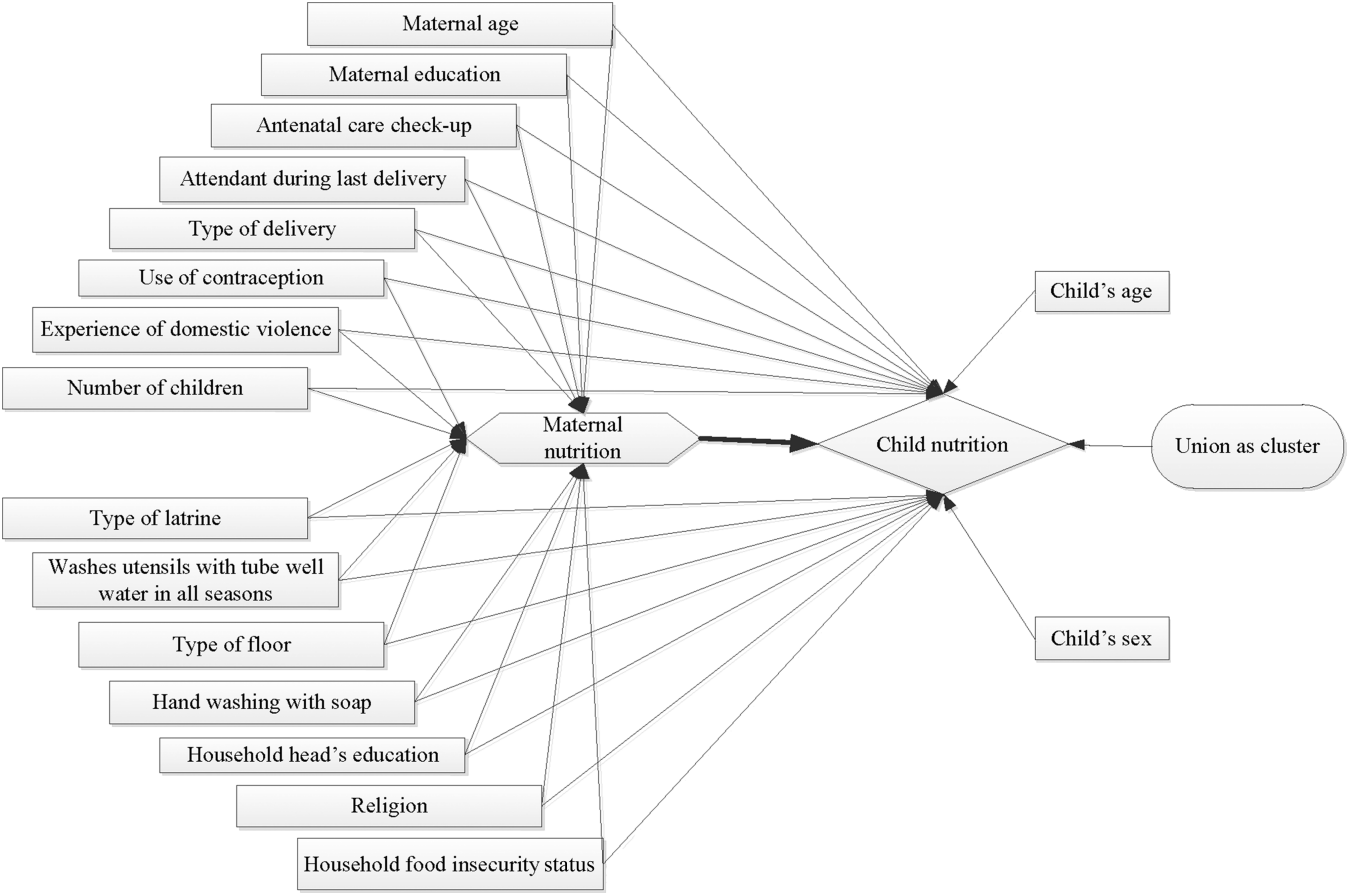
Conceptual framework of factors associated with maternal and child nutritional status

The conceptual framework of factors influencing the association between maternal undernutrition and child nutrition is depicted in Figure 1. The outcome variables for this study were indicators of child nutritional status, such as wasting, stunting and underweight. The length of children was measured using seca 416 infantometers (seca, Hamburg, Germany) with a precision of 0.1 cm. Seca 874 weight scales with a precision of 1 gm were used to measure maternal and child weight. Maternal MUAC was measured at the mid‐point between the tip of the shoulder and the tip of the elbow (olecranon process and acromion process; Mehranfar et al., 2020). The left arm was bent to a right angle, the wide end of the tape was placed on the shoulder and the length to the elbow was measured. The midpoint of the left upper arm was determined (and ideally, marked with a pen). Then, the arm was straightened, and the tape was placed at the mid‐point to measure MUAC, ensuring the tape was not too tight or too loose. Three readings were taken, and the average was computed using commands in the database. Low maternal MUAC was defined as a MUAC < 23 cm, which correlates closely with low BMI (Ghosh et al., 2019; Sultana et al., 2015). Length‐for‐age (LAZ), weight‐for‐age (WAZ) and weight‐for‐length (WLZ) *Z* scores were calculated as indicators of child nutritional status according to the 2006 WHO Standards For Children, using *Z*‐score scale = (observed value − average value of the reference population)/standard deviation value of reference population (Haque et al., 2019; Onis & Bloessner, 1997). Stunting was defined as a LAZ < −2, wasting as a WLZ < −2 and underweight as a WAZ < −2.

Several variables were considered as explanatory variables based on an extensive literature review and previous descriptive studies. The variables relevant to maternal nutrition included current age, age at marriage and first pregnancy, formal schooling (at least 1 year), at least four antenatal care check‐up visits during last pregnancy, attendance by qualified health personnel during last delivery, use of contraceptives, type of delivery (vaginal or caesarean section), experience of domestic violence and handwashing with soap and water before eating food and after defecation or handling domestic animals. The household demographic variables included household size, the sex and education of the household head, religion, type of latrine (improved or non‐improved), washing utensils with tube well water in all seasons, type of floor (improved or non‐improved) and household food insecurity status.

Maternal experience of domestic violence was defined as a husband threatening to divorce or remarry, or verbal or physical abuse by their husband or other family members (Haque et al., 2020). The Household Food Insecurity Access Scale (HFIAS) was employed to define food insecurity according to the Food and Nutrition Technical Assistance's guidelines and categorized as food secure, mildly food insecure, moderately food insecure or severely food insecure (Coates et al., 2007; Haque et al., 2017).

### 2.3 Quality control

The team supervisors were responsible for responding to critical questions on use of the Android tablets and also verified that the respondents from non‐responsive households were truly unavailable or had opted out of participation. The precision of all instruments was checked every morning, and weighing scales were calibrated daily. The supervisors cleaned all instruments before going to the field sites, and the anthropometric team cleaned the instruments carefully after each use. In addition to the data collection team, an independent quality control team of four quality control officers made random visits to the data collection teams to observe the data collection, sampling and re‐interviewing processes for a randomly selected sub‐sample of respondents.

### 2.4 Statistical analysis

All statistical analyses were performed using Stata 14 (StataCorp, College Station, TX, USA). Data were visualized using box plots, histogram, pie charts, bar diagrams or scatter plots. Descriptive statistics such as the mean/standard deviations for quantitative variables and frequencies/proportions for qualitative variables were used to summarize the data and segregated by maternal nutritional status. The chi‐square test was used to assess the association between two categorical variables; independent *t* tests were used to determine the significance of the differences between the means of two groups for normally distributed data. Primarily, simple logistic regression analysis was used to establish the strength of associations between low maternal MUAC and other variables. Multiple logistic regression analysis was employed to identify the factors potentially associated with maternal underweight status and assess the association between maternal underweight status and indicators of child nutritional status after controlling for union as a cluster. Covariates with a *p* < 0.25 in the bivariate analysis were included in the multiple regression model via a stepwise forward selection method (Bursac et al., 2008; Haque et al., 2020). *P* < 0.05 was considered as the level of significance, and 95% confidence intervals were calculated to assess the directions and strength of the effects.

### 2.5 Ethical considerations

This study was approved by the Research Review Committee and Ethical Review Committee, the two obligatory components of the institutional review board of icddr,b. Informed written consent was obtained from study participants.

## 3 RESULTS

**FIGURE 2:**
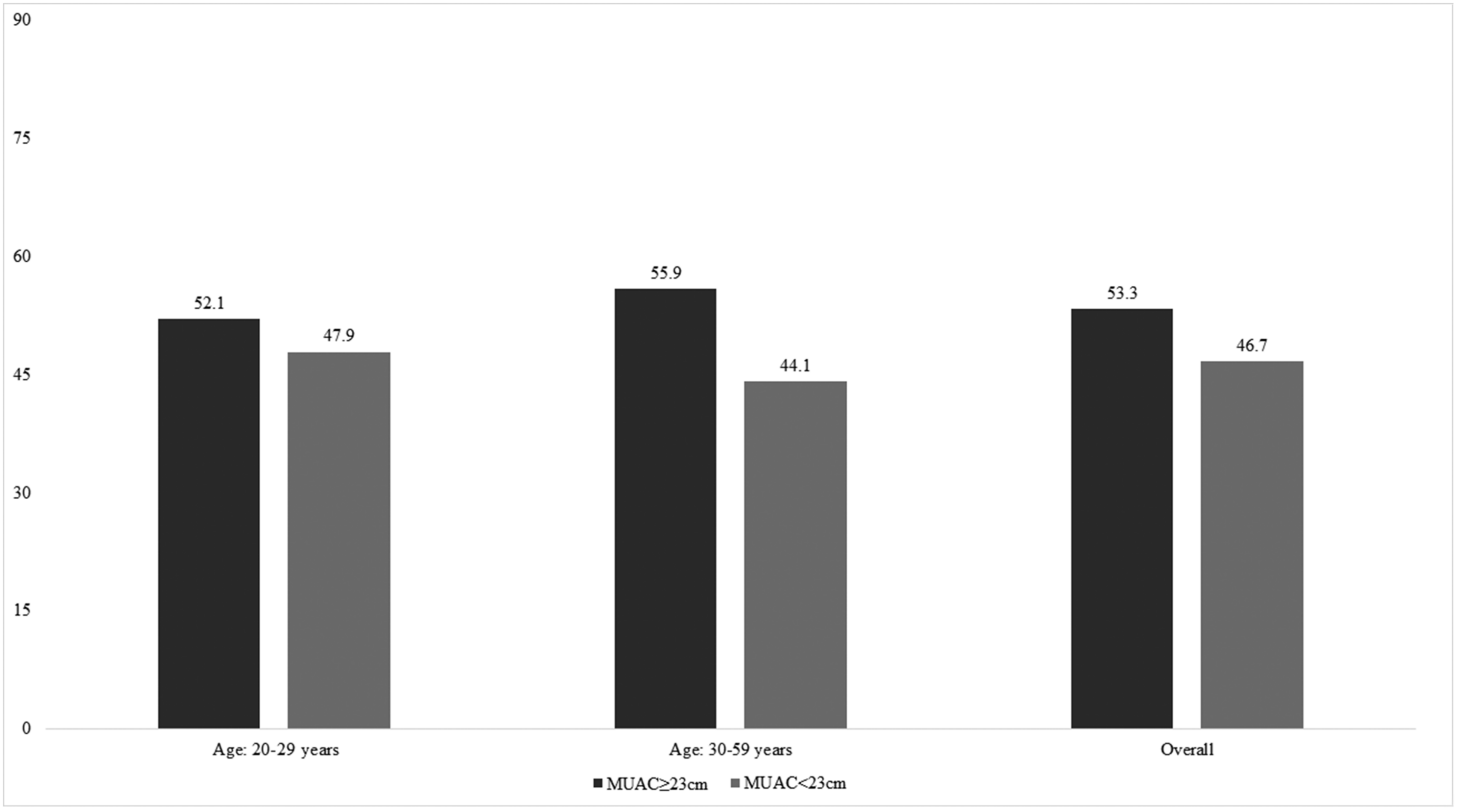
Age‐stratified prevalence of maternal underweight status based on a maternal low mid‐upper arm circumference <23 cm

A total of 7,723 households were visited. Of these, 2,283 households were not surveyed for various reasons (Figure S1). Moreover, 371 households were not included in the analysis, as maternal age was <20 years, because this adolescent age still has potential to grow further. In total, 5,069 households with mother–child pairs were analysed in this study. Overall, 2,367 (47%) mothers had a MUAC < 23 cm (Figure 2). Among the mothers with a low MUAC, the prevalence of stunting among their children was 48.8%, wasting was 13.4% and underweight was 38.9%. The descriptive findings for this dataset segregated by maternal nutritional status are shown in Table 1.

**TABLE 1:** General characteristics of the participants

	Maternal mid‐upper arm circumference (MUAC)		
	MUAC ≥ 23 cm	MUAC < 23 cm	Overall
Indicator	*n* = 2,702	*n* = 2,367	*n* = 5,069
Child's characteristics			
Sex			
Female	50.19 (1,356)	48.12 (1,139)	49.22 (2,495)
Male	49.81 (1,346)	51.88 (1,228)	50.78 (2,574)
Age in months			
0–5	18.39 (497)	17.74 (420)	18.09 (917)
6–11	21.1 (570)	22.1 (523)	21.56 (1,093)
12–23	60.51 (1,635)	60.16 (1,424)	60.35 (3,059)
Consumed colostrum			
No	12.03 (325)	18.59 (440)	15.09 (765)
Yes	87.97 (2,377)	81.41 (1,927)	84.91 (4,304)
Age‐appropriate infant and young child feeding practices			
No	10.25 (277)	11.53 (273)	10.85 (550)
Yes	89.75 (2,425)	88.47 (2,094)	89.15 (4,519)
Length‐for‐age Z‐score^a^	−1.71 ± 1.26	−1.98 ± 1.33	−1.84 ± 1.3
Weight‐for‐age Z‐score^a^	−1.33 ± 1.13	−1.73 ± 1.17	−1.52 ± 1.16
Weight‐for‐length Z‐score^a^	−0.53 ± 1.12	−0.88 ± 1.13	−0.69 ± 1.14
Stunting	40.45 (1,093)	48.80 (1,155)	44.35 (2,248)
Wasting	7.85 (212)	13.43 (318)	10.46 (530)
Underweight	25.68 (694)	38.91 (921)	31.86 (1,615)
Maternal characteristics			
Age in years			
30–59	33.57 (907)	30.21 (715)	32.00 (1,622)
20–29	66.43 (1,795)	69.79 (1,652)	68.00 (3,447)
Maternal education			
No schooling	21.69 (586)	26.66 (631)	24.01 (1,217)
Primary incomplete	21.47 (580)	22.73 (538)	22.06 (1,118)
Primary complete or above	56.85 (1,536)	50.61 (1,198)	53.94 (2,734)
Antenatal care check‐ups during last pregnancy			
Less than four	84.27 (2,277)	89.44 (2,117)	86.68 (4,394)
At least four	15.73 (425)	10.56 (250)	13.32 (675)
Attendant during last delivery			
Qualified	35.23 (952)	27.21 (644)	31.49 (1,596)
Unqualified	64.77 (1,750)	72.79 (1,723)	68.51 (3,473)
Type of delivery			
Vaginal	87.05 (2,352)	92.82 (2,197)	89.74 (4,549)
Caesarean	12.95 (350)	7.18 (170)	10.26 (520)
Received post‐natal care			
No	64.15 (1,709)	69.92 (1,634)	66.85 (3,343)
Yes	35.85 (955)	30.08 (703)	33.15 (1,658)
Number of children			
1	16.28 (440)	18.29 (433)	17.22 (873)
2–3	44.49 (1,202)	44.36 (1,050)	44.43 (2,252)
4+	39.23 (1,060)	37.35 (884)	38.35 (1,944)
Use of contraception			
No	40.08 (1,083)	52.56 (1,244)	45.91 (2,327)
Yes	59.92 (1,619)	47.44 (1,123)	54.09 (2,742)
Experience of domestic violence			
No	67.25 (1,817)	62.10 (1,470)	64.85 (3,287)
Yes	32.75 (885)	37.90 (897)	35.15 (1,782)
Household characteristics			
Type of latrine			
Improved	38.45 (1,039)	30.33 (718)	34.66 (1,757)
Non‐improved	61.55 (1,663)	69.67 (1,649)	65.34 (3,312)
Water and soap available in handwashing place			
No	68.76 (1,858)	77.65 (1,838)	72.91 (3,696)
Yes	31.24 (844)	22.35 (529)	27.09 (1,373)
Wash utensils with tube well water in all seasons			
No	51.78 (1,399)	60.12 (1,423)	55.67 (2,822)
Yes	48.22 (1,303)	39.88 (944)	44.33 (2,247)
Religion			
Non‐Muslim	6.74 (182)	11.33 (268)	8.89 (450)
Muslim	93.26 (2,517)	88.67 (2,097)	91.11 (4,614)
Type of floor			
Improved	13.66 (369)	7.44 (176)	10.75 (545)
Non‐improved	86.34 (2,333)	92.56 (2,191)	89.25 (4,524)
Household food insecurity status			
Food secure	15.91 (430)	11.79 (279)	13.99 (709)
Mildly food insecure	11.99 (324)	9.17 (217)	10.67 (541)
Moderately food insecure	45.23 (1,222)	47.95 (1,135)	46.5 (2,357)
Severely food insecure	26.87 (726)	31.09 (736)	28.84 (1,462)
Household size			
≤7	74.32 (2,008)	76.05 (1,800)	75.12 (3,808)
>7	25.68 (694)	23.95 (567)	24.88 (1,261)
Source of drinking water			
Other	11.32 (306)	16.18 (383)	13.59 (689)
Tube well	88.68 (2,396)	83.82 (1,984)	86.41 (4,380)

^a^ Mean and standard deviation.

The adjusted and unadjusted odds ratios computed from logistic regression are shown in Table 2. The factors significantly associated with maternal undernutrition based on MUAC were a maternal age of 20–29 [vs. 30–59‐years‐old; aOR: 1.24 (95% CI: 1.08, 1.42); *p* = 0.002], less than four antenatal care check‐ups during last pregnancy [aOR: 1.33 (95% CI: 1.10, 1.61); *p* = 0.004], attended by an unqualified birth attendant during last delivery [aOR: 1.29 (95% CI: 1.13, 1.47); *p* < 0.001], having a single child [vs. multiple children; aOR: 1.21 (95% CI: 1.03, 1.44); *p* = 0.024], no use of contraceptives [aOR: 1.56 (95% CI: 1.41, 1.74); *p* < 0.001], maternal experience of domestic violence [aOR: 1.20 (95% CI: 1.07, 1.34); *p* = 0.001], use of non‐improved latrines [aOR: 1.16 (95% CI: 1.02, 1.30); *p* = 0.019], the household not having water and soap in their handwashing place [aOR: 1.27 (95% CI: 1.10, 1.46); *p* = 0.001], religious status other than Muslim [aOR: 1.96 (95% CI: 1.56, 2.45); *p* < 0.001], moderately/severely insecure households [vs. food secure/mildly insecure; aOR: 1.23 (95% CI: 1.06, 1.42); *p* = 0.007], not washing utensils with tube well water in all seasons [aOR: 1.23 (95% CI: 1.08, 1.40); *p* = 0.002] and having an unimproved floor as a construction material [aOR: 1.40 (95% CI: 1.14, 1.73); *p* = 0.002].

**TABLE 2:** Factors associated with maternal underweight

Indicator	Unadjusted OR		Adjusted OR	
	(95% CI)	*P* value	(95% CI)	*P* value
Maternal age in years				
30–59	Reference		Reference	Reference
20–29	1.17 (1.03, 1.33)	0.017	1.24 (1.08, 1.42)	0.002
Antenatal care check‐up during the last pregnancy				
At least four	Reference		Reference	Reference
Less than four	1.58 (1.31, 1.91)	0.000	1.33 (1.10, 1.61)	0.004
Attendant during last delivery				
Qualified	Reference		Reference	Reference
Unqualified	1.46 (1.29, 1.64)	0.000	1.29 (1.13, 1.47)	0.000
Number of children				
Multiple	Reference		Reference	Reference
Single	1.15 (0.98, 1.35)	0.077	1.21 (1.03, 1.44)	0.024
Use of contraception				
Yes	Reference		Reference	Reference
No	1.66 (1.48, 1.86)	0.000	1.56 (1.41, 1.74)	0.000
Maternal experience of domestic violence				
No	Reference		Reference	Reference
Yes	1.25 (1.12, 1.40)	0.000	1.20 (1.07, 1.34)	0.001
Type of latrine				
Improved	Reference		Reference	Reference
Unimproved	1.43 (1.27, 1.62)	0.000	1.16 (1.02, 1.30)	0.019
Water and soap available in handwashing place				
Yes	Reference		Reference	Reference
No	1.58 (1.39, 1.79)	0.000	1.27 (1.10, 1.46)	0.001
Religion				
Muslim	Reference		Reference	Reference
Non‐Muslim	1.77 (1.38, 2.26)	0.000	1.96 (1.56, 2.45)	0.000
Household food insecurity status				
Food secure/mildly insecure	Reference		Reference	Reference
Moderately/severely insecure	1.46 (1.27, 1.68)	0.000	1.23 (1.06, 1.42)	0.007
Washes utensils with tube well water in all seasons				
Yes	Reference		Reference	Reference
No	1.40 (1.23, 1.61)	0.000	1.23 (1.08, 1.40)	0.002
Type of floor material				
Improved	Reference		Reference	Reference
Non‐improved	1.97 (1.60, 2.43)	0.000	1.40 (1.14, 1.73)	0.002

*Note:* Union was adjusted as a cluster.

After adjusting for covariates (maternal age in years, received post‐natal care [PNC] visits after last delivery, type of latrine, type of floor, household food insecurity status, household size, source of drinking water, child's sex, child's age, whether the child was given colostrum and age‐appropriate breastfeeding) and union as a cluster in the multiple logistic regression model, maternal MUAC < 23 cm was associated with wasting [OR: 1.78 (95% CI: 1.5, 2.12); *p* < 0.001], underweight [OR: 1.77 (95% CI: 1.61, 1.96); *p* < 0.001] and stunting [OR: 1.32 (95% CI: 1.19, 1.47); *p* < 0.001] among children (Table 3).

**TABLE 3:** Association of maternal low MUAC with childhood wasting, underweight and stunting

	Wasting		Underweight		Stunting	
	Adjusted OR (95% CI)	*P* value	Adjusted OR (95% CI)	*P* value	Adjusted OR (95% CI)	*P* value
Maternal nutritional status						
Underweight (MUAC < 23 cm)	Reference		Reference		Reference	
Normal (MUAC ≥ 23 cm)	1.78 (1.5, 2.12)	0.000	1.77 (1.61, 1.96)	0.000	1.32 (1.19, 1.47)	0.000
Maternal age in years						
30–59	Reference		Reference		Reference	
20–29	1.25 (1.04, 1.5)	0.019	1.46 (1.27, 1.67)	0.000	1.24 (1.1, 1.40)	0.000
Had post‐natal care visits after last delivery						
Yes	Reference		Reference		Reference	
No	1.22 (0.99, 1.51)	0.060	1.31 (1.15, 1.50)	0.000	1.27 (1.13, 1.44)	0.000
Type of latrine						
Improved	Reference		Reference		Reference	
Non‐improved	1.34 (1.08, 1.66)	0.007	1.19 (1.02, 1.37)	0.024	1.20 (1.06, 1.36)	0.004
Type of floor						
Improved	Reference		Reference		Reference	
Non‐improved	0.78 (0.56, 1.09)	0.148	1.25 (1.00, 1.56)	0.048	1.26 (1.03, 1.55)	0.024
Household food insecurity status						
Food secure/mildly insecure	Reference		Reference		Reference	
Moderately/severely insecure	1.04 (0.82, 1.34)	0.731	1.23 (1.08, 1.41)	0.002	1.33 (1.19, 1.48)	0.000
Household size (HH size)						
≤ 7	Reference		Reference		Reference	
> 7	1.11 (0.88, 1.40)	0.364	1.24 (1.09, 1.42)	0.002	1.09 (0.97, 1.24)	0.158
Source of drinking water						
Other	Reference		Reference		Reference	
Tube well	1.07 (0.81, 1.41)	0.649	1.08 (0.89, 1.30)	0.441	1.22 (1.05, 1.42)	0.010
Child's sex						
Female	Reference		Reference		Reference	
Male	1.29 (1.08, 1.55)	0.006	1.28 (1.11, 1.48)	0.001	1.29 (1.14, 1.46)	0.000
Child's age in months						
0–5	Reference		Reference		Reference	
6–11	2.02 (1.46, 2.80)	0.000	2.01 (1.64, 2.46)	0.000	2.73 (2.24, 3.34)	0.000
12–23	1.67 (1.14, 2.47)	0.009	1.20 (0.94, 1.53)	0.151	1.24 (1.01, 1.54)	0.042
Child was given colostrum						
Yes	Reference		Reference		Reference	
No	1.16 (0.95, 1.41)	0.147	1.17 (1.01, 1.35)	0.042	1.11 (0.95, 1.30)	0.171
**Age‐appropriate breastfeeding**						
Yes	Reference		Reference		Reference	
No	1.57 (1.10, 2.22)	0.012	1.23 (0.98, 1.54)	0.073	1.10 (0.88, 1.38)	0.397

*Note:* Union was adjusted as a cluster.

## 4 DISCUSSION

This study examined data collected during the baseline survey of the Suchana programme to identify factors significantly associated with maternal underweight status, as indicated by MUAC, and indicators of child nutritional status. After controlling for many relevant covariates, which were available in our data set, maternal MUAC < 23 cm was significantly correlated with wasting, stunting and underweight status among children. These findings are consistent with results from other settings (Assefa et al., 2012; Gewa et al., 2012; Kpewou et al., 2020; Vasundhara et al., 2020), suggesting that maternal MUAC is a significant predictor of childhood malnutrition.

Our analysis revealed that younger maternal age, not receiving antenatal care check‐ups during the last pregnancy, not having a skilled attendant during the last delivery, the number of children, not using of contraception, experience of domestic violence, having unimproved latrine, no availability of water and soap in the handwashing place, religion, household food insecurity, not washing utensils with tube well water in all seasons and having unimproved flooring material were significantly associated with maternal underweight status. The associations between maternal underweight status and general characteristics such as age, education and the number of children are broadly consistent with the observations of previous studies in Bangladesh (Balarajan & Villamor, 2009; Hasan et al., 2017; Kamal et al., 2015; Khan & Kraemer, 2009). Similarly, the results of several nationally representative studies indicated that mothers belonging to households in low socio‐economic strata are significantly more vulnerable to being underweight (Balarajan & Villamor, 2009; Khan & Kraemer, 2009; Oddo et al., 2012). These studies used the household wealth index as an indicator of socio‐economic status, which reflects a number of important indicators of economic status such as the type of latrine, source of water and construction material. We found a large number of socio‐economic indicators were significantly associated with maternal underweight status, in agreement with other studies conducted in India and Cambodia (Chattopadhyay et al., 2019; Janmohamed et al., 2016; M. F. Young et al., 2020). However, as far as we are aware, those studies did not assess the indicators simultaneously in a single model after controlling for other important indicators.

Several hypotheses may be proposed to explain the factors associated with maternal underweight status. For instance, having a non‐improved toilet facility, non‐improved floor, using tube well water to wash utensils, and handwashing without soap before taking food or after defecation or nurturing domestic animals increase the chances of exposure to pathogens. Being underweight is linked to infections in both developing and developed countries (Katona & Katona‐Apte, 2008). Moreover, faecal contagion during children's play and feeding children in polluted environments have been reported as constant, cumulative health risks during the vital window of child growth and development (Ngure et al., 2014). Our findings of moderate and severe food insecurity at the household level and the significant association between household food insecurity and maternal undernutrition are in agreement with earlier reports (Kamal et al., 2015) that these variables are predictors of malnutrition in children (Choudhury et al., 2017; Corsi et al., 2011). In terms of the maternal health outcomes associated with undernutrition in this study, receiving ANC during pregnancy, the type of birth attendant during the last delivery and contraceptive use have been widely scrutinized as factors that contribute to both maternal and child health (Choudhury et al., 2017; Perry et al., 2017; Rahman et al., 2016; Seward et al., 2017; Siddique et al., 2018).

This study suggests that a better understanding of several factors—including water, sanitation and hygiene (WASH), household food insecurity and indicators of maternal healthcare practice—is essential to design appropriate interventions to reduce maternal underweight status. Moreover, the strong correlation between maternal nutrition and growth of the children supports the need to invest more on maternal nutrition to achieve positive changes in children's nutritional status. This study was based on MUAC data, which were obtained carefully by trained analysts. MUAC measurements are less costly, simpler, easier‐to‐obtain and more precise than other anthropometry tools. Thus, MUAC tapes could be useful in other low‐resource settings where precise weight and height scales would be expensive. Furthermore, mothers or other household members can receive appropriate training in measuring MUAC from local healthcare providers, such as community clinics, family welfare centres or Upazila health complexes. Thus, mothers or household member or local healthcare providers could measure MUAC to easily assess current nutritional status and encourage adolescents and pregnant or lactating women within the community to adopt healthier lifestyle. Assessment of maternal MUAC may also help to predict the future nutrition status of newborn babies and enable the necessary initiatives to be taken to reduce undernutrition in the corresponding household. In the broader context, MUAC could also be employed at the national level to assess maternal and child nutritional status in population surveillance programmes.

We used the globally recommended cut‐off value of 23 cm for MUAC; however, it is uncertain whether this cut‐off is appropriate for this resource‐poor setting. Additional research is necessary to determine the optimal cut‐off to establish maternal MUAC as a predictor of child nutritional status. Moreover—as a major limitation of maternal MUAC—if the mother is an adolescent, that is, still growing and developing, their MUAC may not be comparable to older women's MUAC; thus, further research is required to identify age‐specific cut‐off values for maternal MUAC.

### 4.1 Strengths and limitations

The strengths of this research include the large sample size and strong methodology, including appropriate sampling techniques, adequate training, precise anthropometry tools and intensive quality control. Though this analysis indicates an association between maternal underweight status based on MUAC and childhood malnutrition, a causal relationship cannot be assumed due to the cross‐sectional design of this study. Moreover, the data may be subject to recall bias; thus, caution is necessary when drawing conclusions, especially for indicators such as household food insecurity, domestic violence and maternal healthcare.

## 5 CONCLUSION

The socio‐economic and demographic characteristics of the study participants and other indicators, including household food security status, pregnancy‐related healthcare and domestic violence, were found to be significantly correlated with maternal underweight, as indicated by MUAC < 23 cm. After controlling for the effects of these indicators, maternal MUAC was significantly associated with indicators of nutritional status among children from vulnerable households in rural Bangladesh. Comprehensive socio‐economic, environmental and educational interventions are required to address this issue. In order to curb undernutrition among individuals living in poor households in rural Bangladesh, policy makers must take the initiative to build a platform to develop multidisciplinary interventions.

## CONFLICTS OF INTEREST

The authors have declared that no conflict of interest exists.

## CONTRIBUTIONS

TA and NC originated the idea for the study and led the protocol design. NC, MJR and SMTA drafted the protocol. SMTA, SSR, MJR, MAH, NC, FDF and TA contributed on survey design. MAH, FDF, ASGF and NC conceptualized the manuscript. MAH performed statistical analysis and drafted the manuscript. MAH and FDF contributed to literature review and selection of variables. NC and ASGF supervised the work and critically reviewed and provided feedback for revising the manuscript. NC oversaw the statistical analysis and suggested necessary improvements from statistical point of view as well as public health perspective. MAH, NC, MJR, FDF, MA, TJS, ASGF, TA and SSR contributed to the revision of the final draft for submission. All authors are responsible for the final content of the manuscript.

6

## Supporting information

**Table S1.***Suchana* inclusion criteria for registration of enrolling as Beneficiary Household
**Figure S1**. *Suchana* Household Trial Profile

## Data Availability

The data that support the findings of this study are available on request from the corresponding author. The data are not publicly available due to privacy or ethical restrictions.
