# Supplementary material for: Determinants of maternal low mid‐upper arm circumference and its association with child nutritional status among poor and very poor households in rural Bangladesh
Source: Matern Child Nutr. 2021 May 20;17(4):e13217. doi: 10.1111/mcn.13217 (PMC8476420; doi:10.1111/mcn.13217)
Supplement: Supplementary file 1 — Table S1.Suchana inclusion criteria for registration of enrolling as Beneficiary Household Figure S1. Suchana Household Trial Profile [file MCN-17-e13217-s001.docx]

**Supplementary Table1.** *Suchana* inclusion criteria for registration of enrolling as Beneficiary Household

| **Beneficiary Household Verification Questions** | **Inclusion criteria** |
| --- | --- |
| ***Step 1*** | If “NO” go ahead for next questions |
| - Households currently participating/member of any livelihood, food security or asset transfer program |  |
| ***Step 2*** | If anyone is “NO” go ahead for next questions |
| - Ability to afford three (3) full meals per day for all family members round the year |  |
| - Households monthly income BDT 7,500 or more |  |
| - Household productive asset value worth BDT 15,000 or more (excluding land, pond and homestead) |  |
| - Ownership of homestead land 10 decimals or more |  |
| - Ownership of cultivable land 50 decimals or more (excluding homestead or pond) |  |
| ***Step 3***   - Households have married women with in child bearing age (15 to 45 years) - Households have pregnant women (including abandoned or widowed woman) | If anyone is ‘Yes’ go ahead for registration of enrolling as Beneficiary Household |
| - Households have 0-23 months old children (including children with disabilities) |  |
| - Households have adolescent girls (15-19 years) |  |


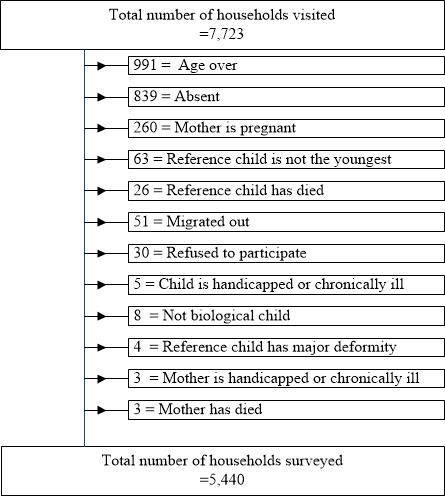


**Supplementary Figure 1**. *Suchana* Household Trial Profile
